# Imaging of patellar fractures

**DOI:** 10.1007/s13244-016-0535-0

**Published:** 2016-11-30

**Authors:** Mohamed Jarraya, Luis E. Diaz, William F. Arndt, Frank W. Roemer, Ali Guermazi

**Affiliations:** 1 Department of Radiology, Mercy Catholic Medical Center, 1500 Lansdowne Avenue, Darby, 19023 PA USA; 2 Department of Radiology, Boston University School of Medicine, Boston, MA USA; 3 Department of Radiology, VA Boston Healthcare System, Boston, MA USA; 4 Department of Radiology, University of Erlangen-Nuremberg, Erlangen, Germany

**Keywords:** Patella, Fracture, Extensor mechanism rupture, Conventional radiograph, MRI

## Abstract

Patellar fractures account for approximately 1% of all skeletal fractures and may result from direct, indirect, or combined trauma. Because of the importance of patellar integrity for knee extension and the risk of associated injury to the extensor mechanism, accurate reporting and description of fracture type is paramount for appropriate management. This pictorial essay aims to review the normal anatomy of the patella, the mechanisms of injury and different types of patellar fractures, with a brief introduction to therapeutic management.

*Teaching Points*

• *Patellar fractures are classified according to their morphology and degree of displacement*.

• *Direct trauma results in stellate fractures*.

• *Indirect trauma results in transverse fractures*.

• *Displacement should raise suspicion for retinacular injury*.

## Introduction

Patellar fractures account for approximately 1% of all skeletal fractures and may result from direct, indirect, or combined injuries [1]. They are most prevalent in individuals between 20 and 50 years of age, and they occur twice as often in men as in women [2]. Patellar fractures are the most common cause of disruption of the extensor mechanism, six times as frequent as soft tissue injuries such as quadriceps or patellar tendon rupture [3]. Fractures may be caused either by excessive force through the extensor mechanism or by a direct blow. Complications include stiffness, extension weakness, and patellofemoral osteoarthritis.

Diagnosis on conventional radiography is usually easy. However, awareness of the different morphologic types and mechanisms of injury is important for subsequent management. Because of the crucial role of the patella in maximizing knee extension, treatment directed toward anatomical restoration is preferred and has been shown to result in improved outcomes [4]. This pictorial essay reviews the relevant anatomy and normal biomechanics of the patella, as well as different mechanisms of injury and types of fractures. An overview of conservative and surgical management is also presented.

### Anatomy and function

**Fig. 1:**
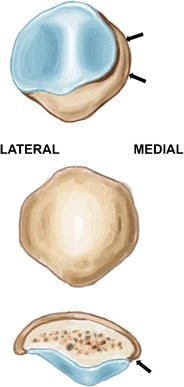
Drawing showing, from top to bottom, the anterior, posterior, and axial views of the patella. The proximal part is termed the basis, and the distal part, the apex. The distal pole is entirely devoid of articular cartilage. A longitudinal ridge divides the cartilaginous surface into medial and lateral facets. The odd facet (*arrows*) is located at the peripheral aspect of the medial facet, and is devoid of cartilage

The patella is the largest sesamoid bone of the body. Despite large variations in shape, the patella is typically ovoid and flat on its anterior non-articular surface. Its proximal margin is termed the basis, and the rounded inferior margin, the apex. The proximal three-fourths of the patella is covered with thick articular cartilage, while the distal pole is entirely devoid of articular cartilage. The proximal articular cartilage is divided into medial and lateral facets by a longitudinal ridge. The peripheral aspect of the medial facet is denuded of cartilage and is termed the odd facet (Fig. 1).

The patella receives its blood supply from an anastomotic ring that originates from the geniculate arteries (the supreme geniculate from the superficial femoral artery, four geniculate arteries arising from the popliteal artery, and the recurrent anterior tibial artery arising from the anterior tibial artery). The superior portion of the ring is located anterior to the quadriceps tendon and the inferior portion is posterior to the patellar tendon. Vessels penetrate the patella from the middle third, and the inferior pole along the infrapatellar fat pad, and perfusion occurs in a distal to proximal fashion [5]. This pattern of retrograde perfusion is important in understanding the risk of osteonecrosis after patellar fracture [1].

The patella is firmly invested within the quadriceps tendon, and belongs to the extensor mechanism, which also includes the quadriceps tendon, medial and lateral retinacula, patella, and patellar tendon. The extensor mechanism is responsible for the extension of the knee and the ability to maintain an erect position. Numerous daily activities such as walking, ascending stairs, and rising from a chair depend on the extensor mechanism to generate sufficient force to overcome gravity. The patella acts as a fulcrum for a mechanical advantage. In fact, without a patella, more force would be required to achieve knee extension [6].

### Imaging modalities

**Fig. 2:**
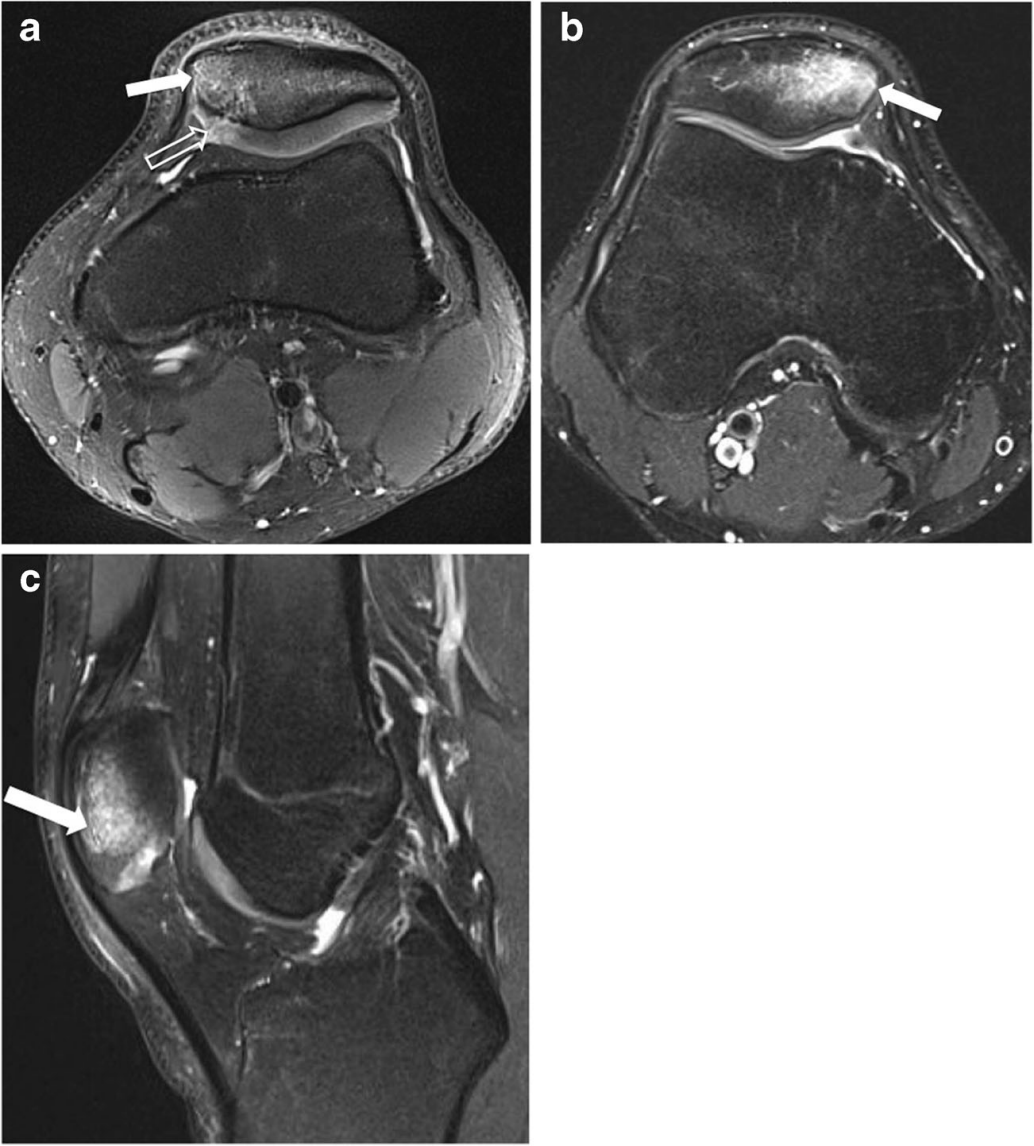
Examples of patellar contusions. (**a**) Axial fat-suppressed proton density-weighted MR image in a 20-year-old man shows hyperintense signal of the medial patellar bone marrow (*solid arrow*) and a fracture line through the adjacent cartilage (*open arrow*). Conventional radiographs were negative for fracture (not shown). (**b**) Axial and (**c**) sagittal fat-suppressed proton density MR images in another 20-year-old man following direct trauma, revealing bone marrow oedema (*arrow*) without cartilage damage. Conventional radiographs were also negative for fracture (not shown)

Evaluation and classification of patellar fractures is based on anteroposterior (AP), lateral, and skyline view radiographs of the knee. A recent study, however, showed that adding computed tomography (CT) to the evaluation led to changes in management plans in almost half of the cases [7]. In that study, CT provided more accurate evaluation of comminuted fractures of the lower pole than did conventional radiography [7]. Magnetic resonance imaging (MRI) is commonly used when a radiographically occult patellar fracture is suspected (Fig. 2). MRI is also highly sensitive for detection of cartilage damage and subchondral fracture and contusion, and provides additional information on the integrity of the soft tissue components of the extensor mechanism.

### Mechanism of injury and classification of patellar fractures

Patellar fractures are the result of direct, indirect, or combined injury [1]. Direct injuries can be secondary to low-energy trauma (fall on the knee from sitting or standing height) or high-energy trauma (dashboard impact in a motor vehicle accident) [1]. Most commonly, though, the mechanism of injury combines direct and indirect patterns, i.e., culmination of a direct blow, quadriceps contraction, and secondary joint collapse [1]. The fracture pattern is not determined solely by the mechanism of injury, but also depends on factors such as patient age, bone quality, and degree of knee flexion [1]. Patellar fractures are commonly classified according to their morphologic pattern and degree of displacement. Melvin and colleagues defined displacement as separation of fracture fragments by more than 3 mm, and/or articular incongruity of more than 2 mm [1].

With indirect injuries, the mechanism of fracture involves failure of the extensor mechanism due to eccentric overload, typically a forceful contraction mechanism of the quadriceps with the knee in flexed position. A classic example is a fall on the feet, in which the quadriceps eccentrically contracts to decelerate the body. When the force of the fall overwhelms the resistance to knee flexion, the extensor mechanism fails, resulting in patellar fracture [8].

### Transverse fractures

**Fig. 3:**
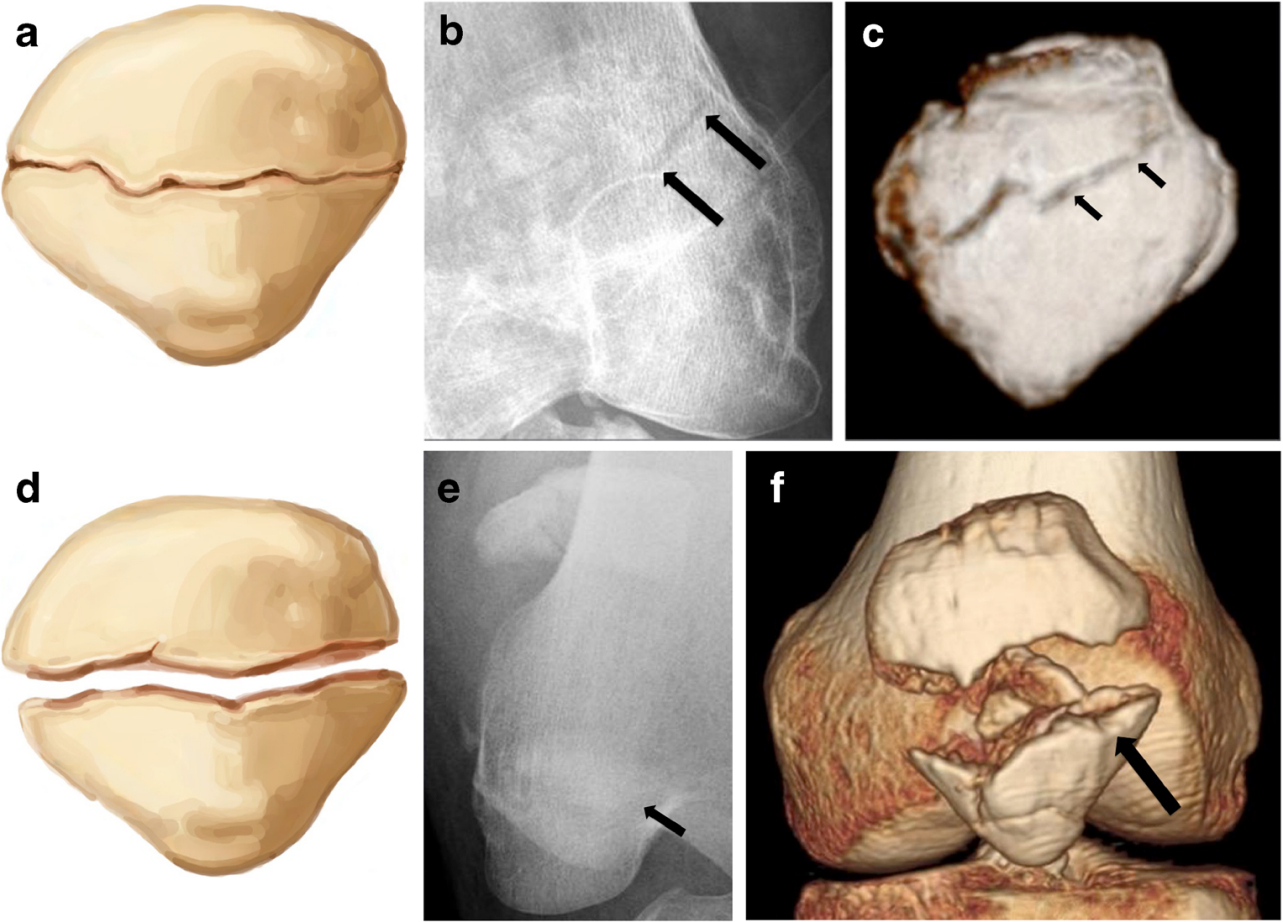
Two examples of transverse fractures in young adults. (**a**, **d**) Drawing, (**b**, **e**) anteroposterior radiograph, and (**c**, **f**) 3D CT surface reconstruction. (**a**–**c**) Non-displaced and (**d**–**f**) displaced transverse fractures through the body of the patella. Displaced fractures are associated with a higher risk of retinacular injury

Approximately 80% of these fractures occur in the middle to lower third of the patella [9]. While the mechanism may be multifactorial, these injuries are typically associated with indirect longitudinal forces [1]. Up to two-thirds of transverse patellar fractures are displaced [10], which should raise suspicion for retinacular and extensor mechanism injury, i.e., tear to the medial and lateral patellar retinacula [1] (Fig. 3).

### Pole fractures

**Fig. 4:**
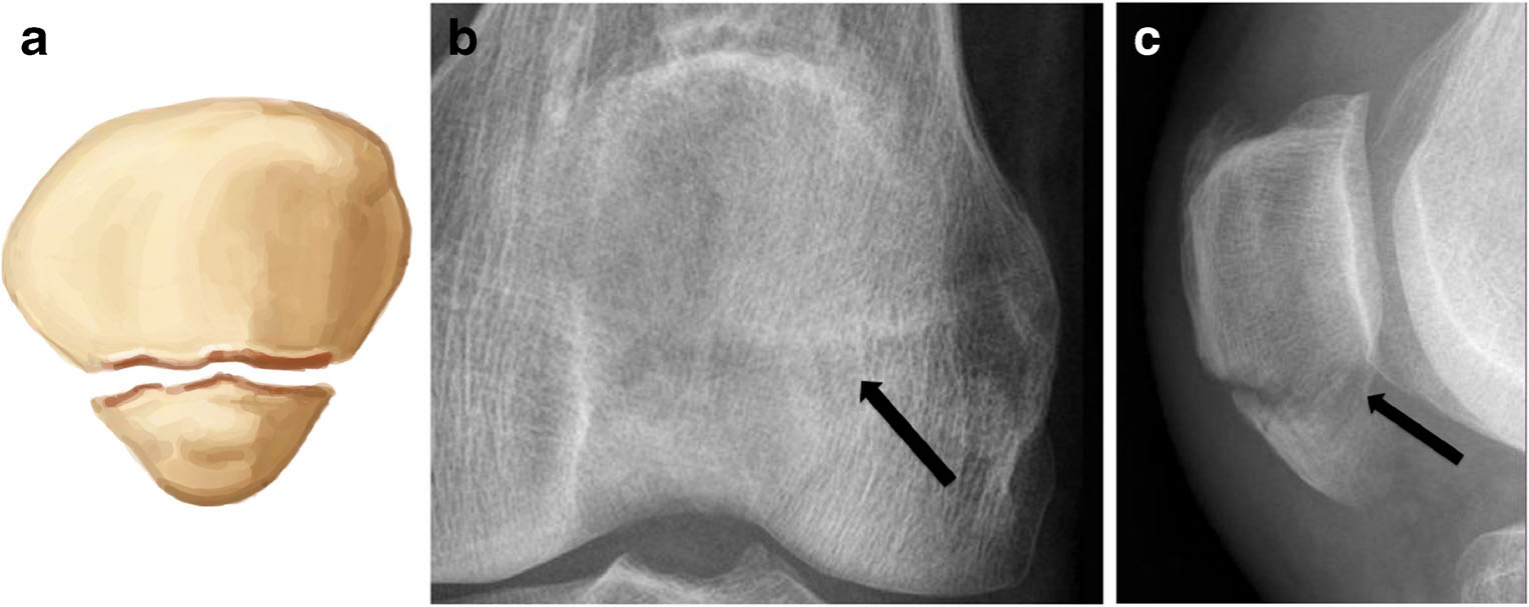
Patellar fracture of the lower pole in a 39-year-old man. (**a**) Drawing and (**b**) anteroposterior and (**c**) lateral radiographs show a transverse fracture of the lower pole of the patella

**Fig. 5:**
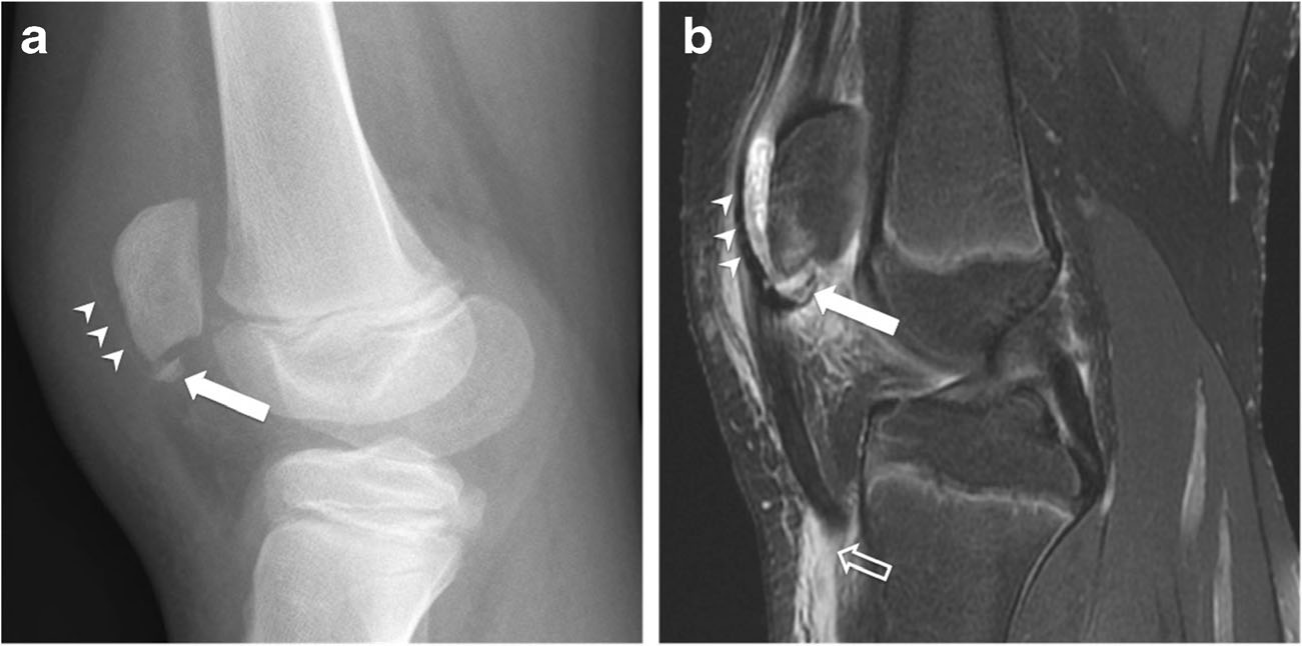
Sleeve fracture of the lower patellar pole in an 11-year-old boy after falling on his flexed knee during a football game, with a popping sound. The patient presented to the ER 5 days later. (**a**) Lateral knee radiograph and (**b**) sagittal fat-suppressed T2-weighted MR image 3 days after knee x-ray show small bony fragment fracture from the lower pole (*arrow*), extending anteriorly, with detachment of a thin shell of cortical bone (*arrowheads*), consistent with a sleeve fracture. Note the injury of the distal attachment of the patellar tendon (*open arrow*). *Images courtesy of Arnold Carlson Merrow, Jr., MD - Cincinnati Children’s Hospital Medical Center, OH, USA*

Small proximal or distal avulsion-type fractures are important to recognize, since they are often associated with substantial soft tissue injury to the quadriceps or patellar tendon [1]. Distal pole fractures (bony avulsion of the patellar tendon) are extra-articular, since the distal pole is devoid of articular cartilage [1]. In this case, lateral radiographs demonstrate patella alta and increased Insall-Salvati ratio [1] (Fig. 4), while upper pole bony avulsions are associated with low-riding patella, also termed patella baja (reduced Insall-Salvati ratio) [1]. In the paediatric population, adolescents are most vulnerable to avulsion fractures because of their increase in muscle strength and relative weakness of osteocartilaginous junctions. In this case, there is a large sleeve of unossified patellar cartilage, which may or may not be accompanied by tiny ossific fragments (Fig. 5). Patellar alignment (alta or baja), location of soft tissue swelling, and joint effusion provide clues to diagnosis [11].

### Stellate fractures

**Fig. 6:**
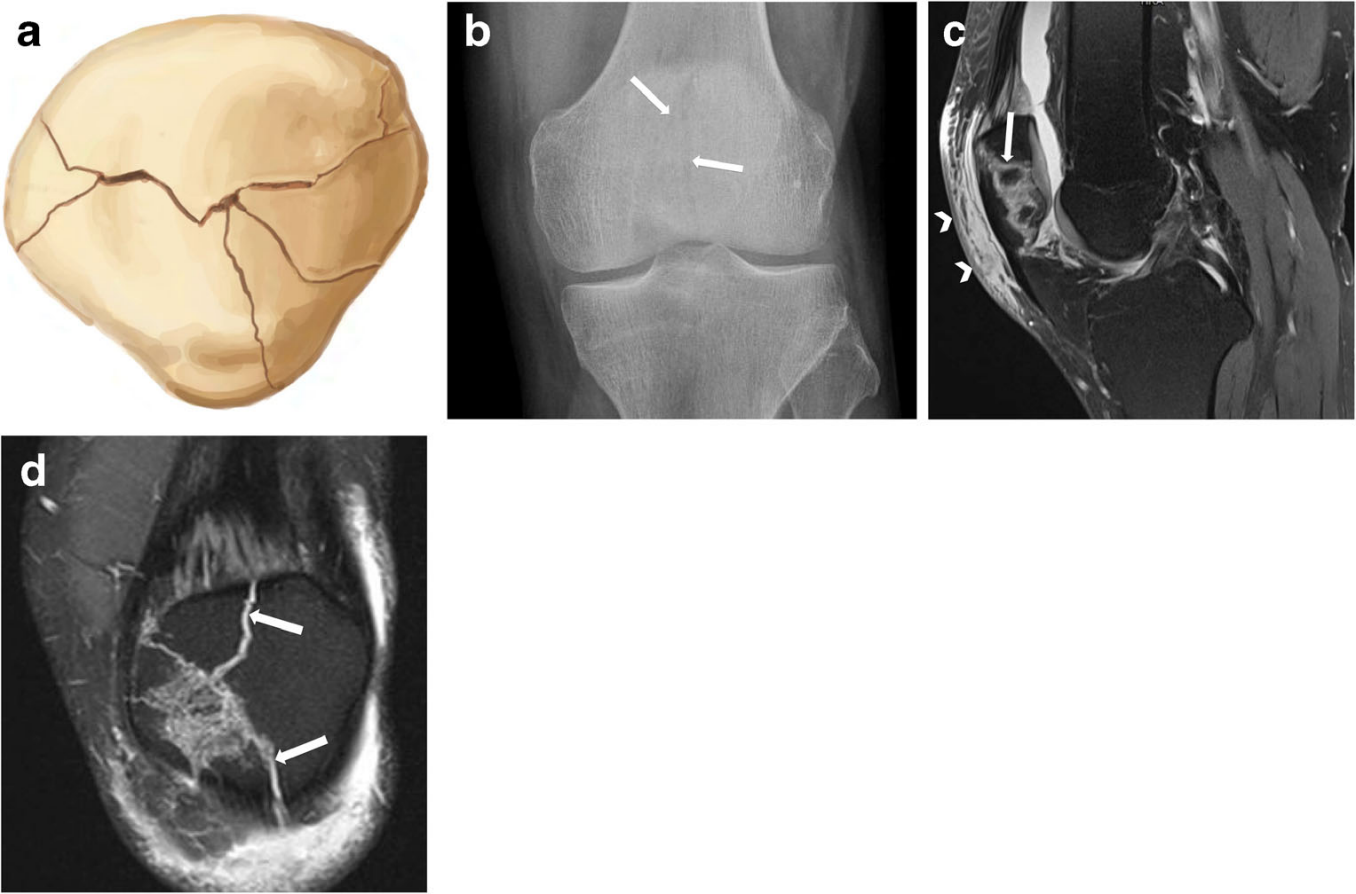
(**a**) Drawing, (**b**) anteroposterior radiograph, and (**c**) sagittal and (**d**) coronal fat-suppressed proton density MR images showing non-displaced stellate fracture (*solid arrows*) in a 36-year-old man. Note the subcutaneous prepatellar oedema related to direct trauma mechanism (*arrowheads*)

**Fig. 7:**
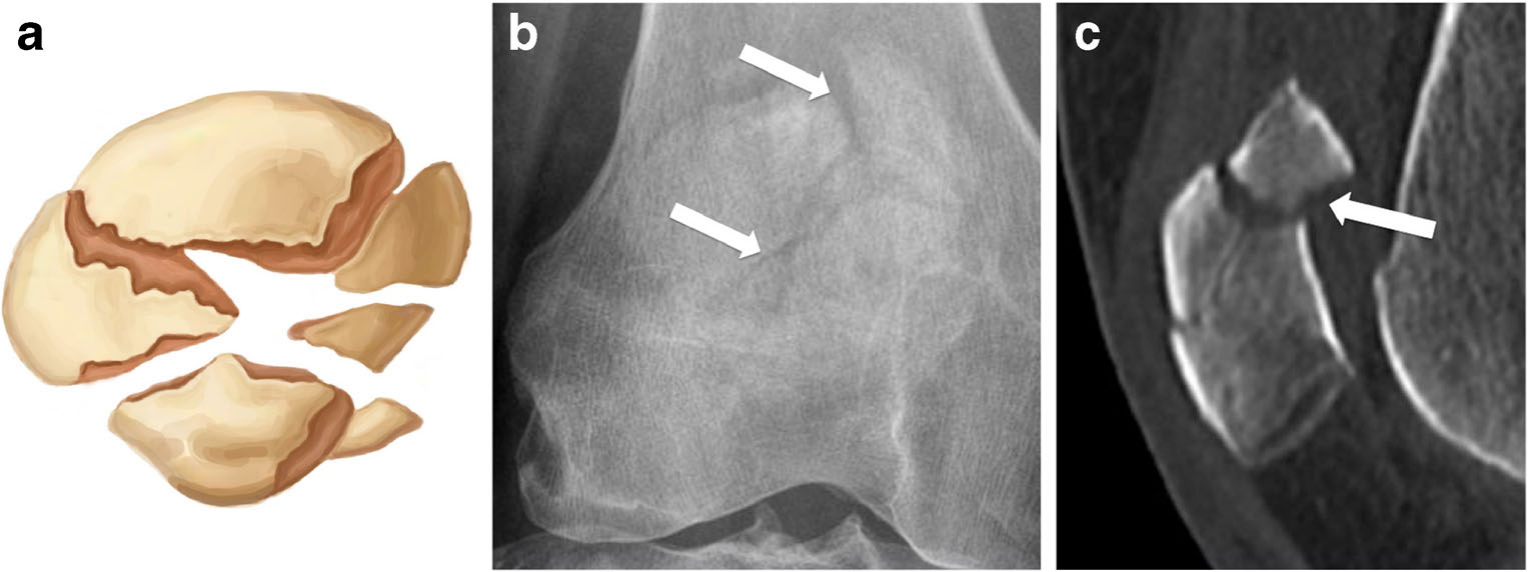
CT reformatted image showing displaced comminuted fracture. (**a**) Drawing, (**b**) anteroposterior radiograph, and (**c**) sagittal computed tomography reformat in a middle-aged man following direct blow to the knee in a car accident, showing displaced comminuted fracture. Note that the degree of displacement is better appreciated on the reformatted CT image than on conventional radiograph

**Fig. 8:**
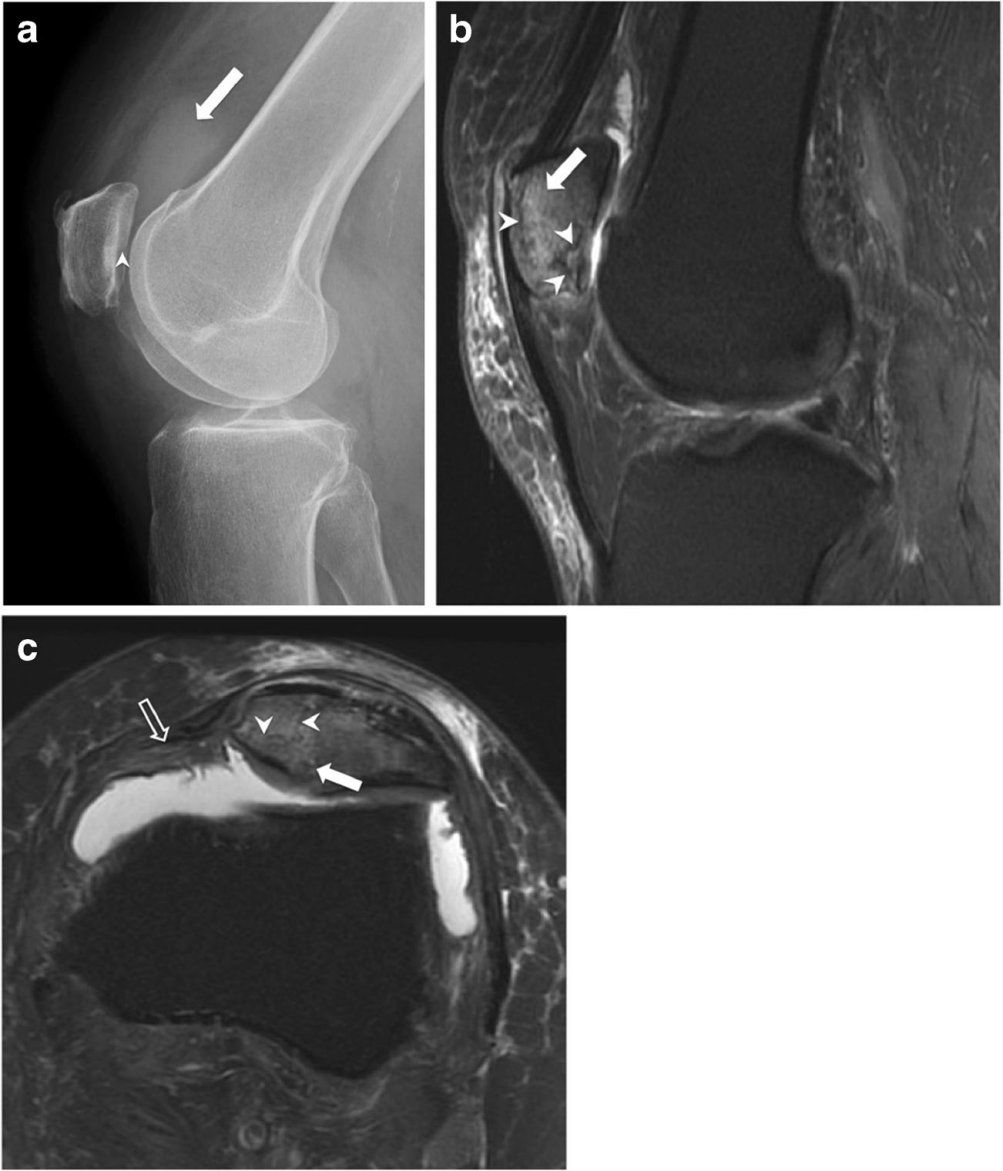
Stellate fracture with retinacular injury in a 40-year-old woman with persistent anterior knee pain for 2 weeks ago after a fall. (**a**) Lateral knee radiograph shows joint effusion (*arrow*) and a subtle linear lucency through the patellar undersurface (arrowhead), suggesting fracture. (**b**) Sagittal and (**c**) axial fat-suppressed proton density MR images reveal several fracture lines (*arrowheads*) consistent with stellate fracture, bone marrow oedema (*solid arrow*), and partial thickness tear of the medial retinaculum from its patellar attachment (*open arrow*)

Stellate fractures result from a direct blow to the patella with the knee in a partially flexed position [1]. Approximately 65% of these injuries are non-displaced [12, 13]. In the case of an extensive comminuted fracture with displacement, the transverse component may extend into the medial and lateral retinacula (Figs. 6, 7, and 8). Associated articular cartilage damage of the patellar and trochlear surfaces is not uncommon [14].

### Vertical fractures

**Fig. 9:**
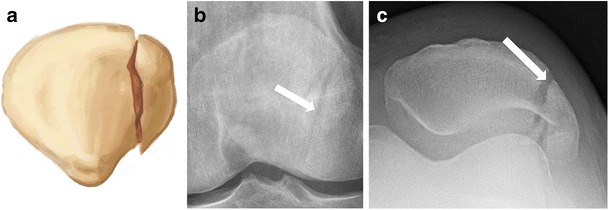
Vertical fracture of the right patella in a 34-year-old man. (**a**) Drawing and (**b**) anteroposterior and (**c**) skyline views of the right knee show a vertical fracture of the lateral facet (*red arrows*). Note that the fracture line is more conspicuous on the tangential than the anteroposterior view

Vertical fractures are also possible, and account for up to 22% of patellar fractures [1]. This type most commonly involves the lateral facet and is thought to result from direct compression of the patella on a hyperflexed knee [15]. Vertical fractures are commonly non-displaced, and the patellar retinacula are usually intact, preserving knee extension. This pattern is easily missed on AP radiographs, emphasizing the importance of a skyline view for identifying this type of injury [1] (Fig. 9).

### Osteochondral fractures

**Fig. 10:**
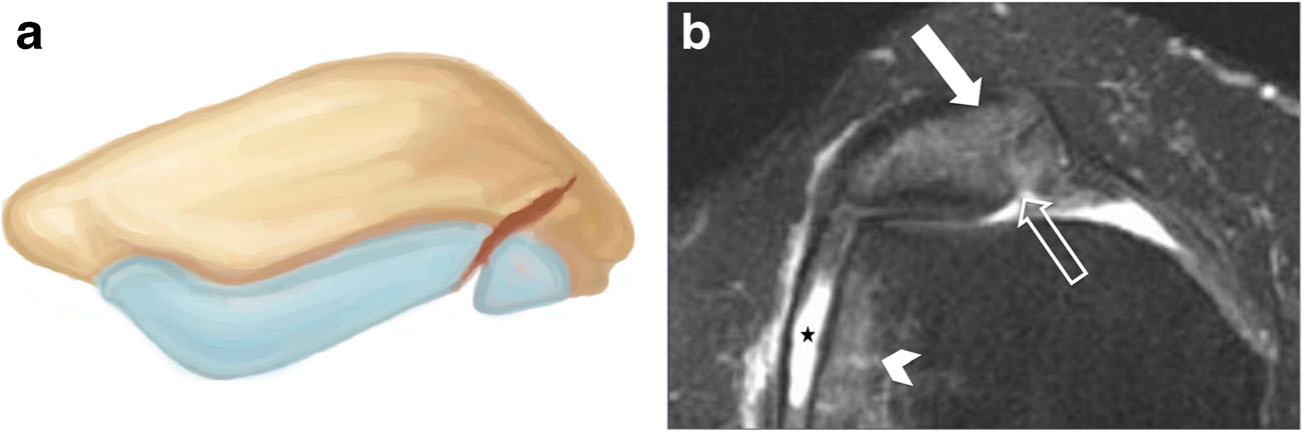
Osteochondral fracture from lateral patellar dislocation in a 27-year-old woman. (**a**) Drawing shows an incomplete osteochondral fracture. (**b**) Axial fat-suppressed proton density MR image shows an osteochondral injury to the articular surface (open arrow) with bone marrow oedema of the medial patella (*solid arrow*) and lateral aspect of the lateral femoral condyle (*arrowhead*), in keeping with transient lateral patellar dislocation. Note also a moderate amount of joint effusion (*star*)

Osteochondral fractures may be seen in association with high-energy stellate patellar fractures or after patellar dislocation. Radiographs may not demonstrate these lesions. Osteochondral fracture fragments can shear from the medial patellar facet or lateral femoral condyle after patellar dislocation/subluxation (Fig. 10) [1].

### Bipartite patella

**Fig. 11:**
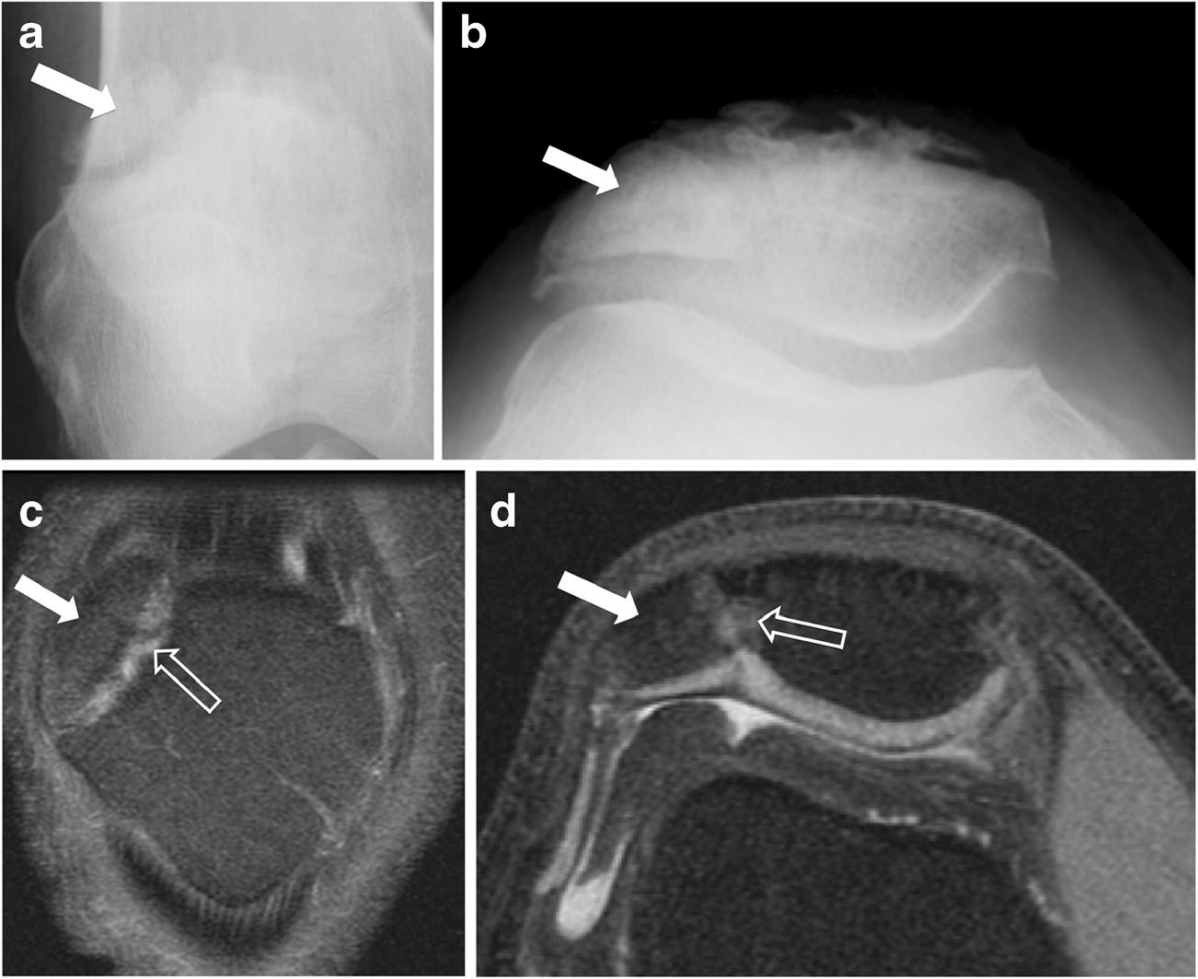
Two examples of incidental bipartite patella. (**a**) Anteroposterior and (**b**) tangential patellar radiographs of the right knee in the first example show a patellar fragment at the superolateral aspect of the right patella consistent with bipartite patella. In the second, (**c**) axial and (**d**) coronal fat-suppressed proton density MR images of the right knee demonstrate a well-corticated osseous fragment (*solid arrow*) at the superolateral aspect of the patella. Note the high signal of the synchondrosis (*open arrows*), the well-preserved underlying cartilage of the patella, and the lack of bone marrow oedema at the adjacent bone that differentiate this variant from a patellar fracture

**Fig. 12:**
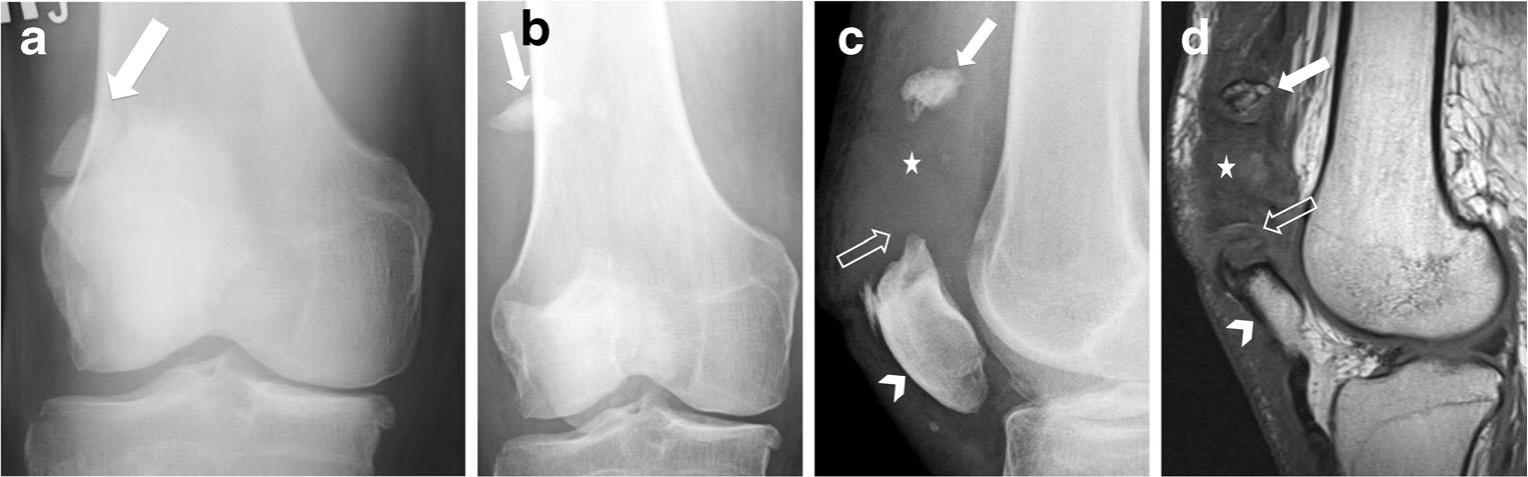
Separation of patella bipartite in a 70-year-old man. (**a**) Anteroposterior radiograph of the right knee demonstrates an incidental bipartite patella (*arrow*). Two years later, the patient presented with anterior knee pain after a fall. (**b**) Anteroposterior and (**c**) lateral radiographs and (**d**) sagittal T1-weighted MR image show separation of the superolateral fragment (*solid arrow*) within the anterolateral soft tissues of the knee, patella baja (*arrowhead*), obliteration of the suprapatellar fat (*open arrow*), and soft tissue swelling and haemorrhage from disruption of the quadriceps tendon (*star*)

An important differential diagnosis of patellar fracture is bipartite/multipartite patella, which results from non-fusion of the accessory patellar ossification centre. The diagnosis is favoured by the location of the patellar fragment in the superolateral aspect of the patella (Fig. 11). Both radiographic appearance and clinical presentation are critical for correct diagnosis. Rarely, a bipartite patella may separate, causing rupture of the extensor mechanism with quadriceps avulsion [16] (Fig. 12).

### Dorsal defect of the patella

Dorsal defect of the patella (DDP) is a common incidental finding on conventional radiographs and MRI during the first decades of life. It is also located at the superolateral quadrant, and is commonly associated with bipartite/multipartite patella [17]. DDP is believed to be an ossification anomaly, possibly stress-induced considering histological evidence of avascular necrosis at the site of the dorsal defect [18, 19]. DDP usually heals spontaneously and is rarely noted in adults [20].

On conventional radiographs, the dorsal defect appears as a round, well-defined radiolucency, with sclerotic margins, located at the dorsal subchondral bone of the superolateral aspect of the patella [18]. The MR signal intensity of the defect usually mirrors that of the overlying cartilage. The cartilage overlying the defect should be closely inspected, as it might fissure and thin [21].

### Management

**Fig. 13:**
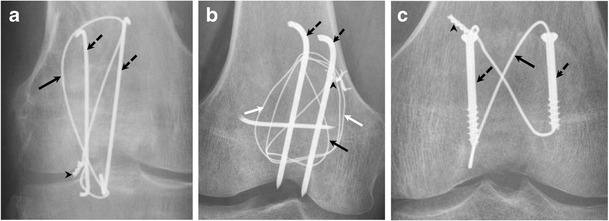
Frontal knee radiographs showing examples of common patellar fracture fixation constructs using tension band wiring. (**a**) Modified anterior tension band (MATB) technique (also called K-wire technique) with vertical figure eight loop configuration and wire twist. (**b**) MATB with vertical figure eight and wire twist in addition to cerclage. (**c**) Cannulated screws with horizontal figure eight loop

**Fig. 14:**
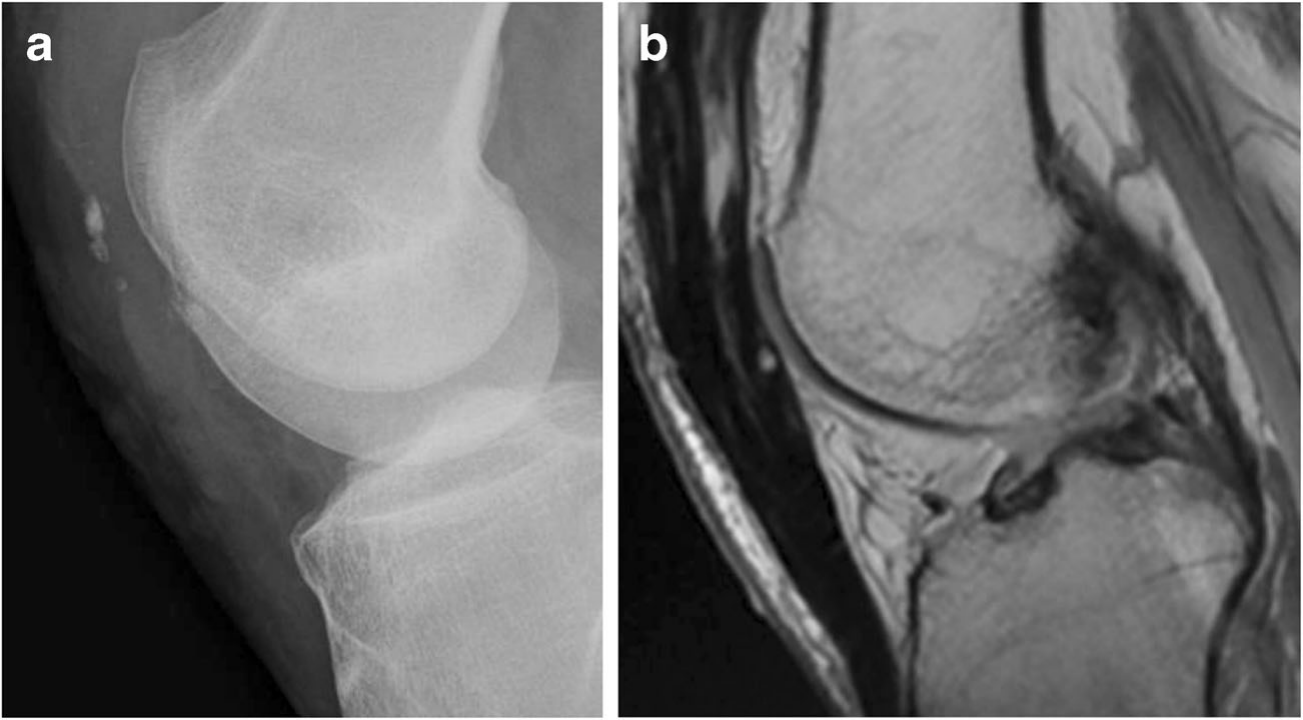
Example of total patellectomy. (**a**) Lateral knee radiograph and (**b**) sagittal proton density MR image of the knee after total patellectomy as a result of surgery following a severely comminuted fracture

Because of the vital mechanical role of the patella in achieving knee extension, the goal of management is directed toward restoring the extensor mechanism while maximizing articular congruency. Non-surgical management is indicated for fractures with a clinically intact extensor mechanism and minimal step-off (<2–3 mm) and/or fracture displacement (<1–4 mm) [12, 22]. Surgical management is indicated in the case of an incompetent extensor mechanism, fracture separation, intra-articular loose bodies, or osteochondral fracture [4]. When surgery is indicated, open reduction with internal fixation may use one or a combination of the following: tension bands, K-wires, cerclage wires, cannulated screws, and fixation plate (Fig. 13) [4]. Partial patellectomy has been described for displaced transverse and comminuted fractures. Retention of a portion of the patella is thought to preserve some of the patellar moment and improve strength [6]. Total patellectomy is indicated in rare cases of failed internal fixation, infection, tumour, or patellofemoral arthritis (Fig. 14) [4].

### Summary

Patellar fractures are rare but can lead to significant clinical deficits, especially when associated with an injury to the extensor mechanism. Diagnosis and accurate classification are important for timely and appropriate management. The most common patellar fractures are transverse, which are secondary to indirect injury, while direct blows typically result in comminuted fractures. Vertical fractures are less common, but may easily be missed on AP radiographs, warranting careful assessment of skyline views. Special care should be taken to examine for displacement, as it may indicate an associated retinacular injury and the need for surgical management. Bipartite patella is an important differential diagnosis of patellar fracture. Management of patellar fractures depends on a number of issues, including clinical presentation and degree of displacement. A wide variety of surgical and non-surgical options are available.
